# The Importance of Lung Ultrasound and IGFBP7 (Insulin-like Growth Factor Binding Protein 7) Assessment in Diagnosing Patients with Heart Failure

**DOI:** 10.3390/jcm13082220

**Published:** 2024-04-11

**Authors:** Anna Szyszkowska, Tomasz Olesiewicz, Izabela Płońska-Korabiewska, Ewa Tarasiuk, Barbara Olesiewicz, Małgorzata Knapp, Rafał Śledziewski, Bożena Sobkowicz, Anna Lisowska

**Affiliations:** 1 Department of Cardiology, Medical University of Bialystok, 15-276 Bialystok, Poland; annaszyszkowska92@gmail.com (A.S.); plonska.izabela@gmail.com (I.P.-K.); ewa-tarasiuk@o2.pl (E.T.); malgo33@interia.pl (M.K.); bozena.sobkowicz@umb.edu.pl (B.S.); 2 Department of Cardiology, Hospital in Ostrów Mazowiecka, 07-300 Ostrów Mazowiecka, Poland; tomasz.olesiewicz7@gmail.com (T.O.); bolesiewicz@wp.pl (B.O.); 3 Department of Radiology, Medical University of Bialystok, 15-276 Bialystok, Poland

**Keywords:** heart failure, HFpEF, HFmrEF, IGFBP7, NTproBNP, lung ultrasound

## Abstract

**Background:** In daily practice, there are problems with adequately diagnosing the cause of dyspnea in patients with heart failure with preserved and mildly reduced ejection fractions (HFpEF and HFmrEF). This study aimed to assess the usefulness of lung ultrasound in diagnosing HFpEF and HFmrEF and determine its correlation with IGFBP7 (insulin-like growth factor binding protein 7), NTproBNP (N-terminal pro–B-type natriuretic peptide), and echocardiographic markers. **Methods:** The research was conducted on 143 patients hospitalized between 2018 and 2020, admitted due to dyspnea, and diagnosed with HFpEF and HFmrEF. Venous blood was collected from all participants to obtain basic biochemical parameters, NTproBNP, and IGFBP7. Moreover, all participants underwent echocardiography and transthoracic lung ultrasound. Two years after hospitalization a follow-up telephone visit was performed. **Results:** The number of B-lines in the LUS ≥ 16 was determined with a sensitivity of—73% and specificity of—62%, indicating exacerbation of heart failure symptoms on admission. The number of B-lines ≥ 14 on admission was determined as a cut-off point, indicating an increased risk of death during the 2-year follow-up period. The factors that significantly impacted mortality in the study patient population were age and the difference between the number of B-lines on ultrasound at admission and at hospital discharge. IGFBP7 levels had no significant effect on the duration of hospitalization, risk of rehospitalization, or mortality during follow-up. **Conclusions:** Lung ultrasonography provides additional diagnostic value in patients with HFpEF or HFmrEF and exacerbation of heart failure symptoms. The number of B-lines ≥ 14 may indicate an increased risk of death.

## 1. Introduction

Heart failure (HF) is caused by a structural and/or functional abnormality of the heart that results in elevated intracardiac pressures and/or inadequate cardiac output at rest and/or during exercise [1]. The most common symptoms are breathlessness, ankle swelling, and fatigue. HF occurs in 1–2% of adults in developed countries, and its prevalence increases with age to >10% in patients aged 70 years or over [1,2,3].

Based on left ventricular ejection fraction, HF is classified into three groups:

- Heart failure with preserved ejection fraction (HFpEF) with EF range ≥ 50%;
- Heart failure with mildly reduced ejection fraction (HFmrEF) with EF range 41–49%;
- Heart failure with reduced ejection fraction (HFrEF) with EF range ≤ 40%.

Data on the distribution of patients in these groups vary depending on the conducted study—according to registers in hospitalized patients, 50% of them have HFrEF and 50% have HFpEF/HFmrEF [1,3,4], and one of the outpatient reports showed that 60% of patients have HFrEF, 24% HFmrEF, and 16% HFpEF [1,5].

Despite significant progress in medicine and new methods of diagnosis and treatment, mortality in patients with HF remains high, and it is up to 67% within the first five years of diagnosis [6]. A better understanding of HF pathophysiology and its early diagnosis may contribute to improved prognosis in this group of patients. Searching for new biomarkers could be one of the solutions.

Insulin-like growth factor binding protein 7 (IGFBP7) is a part of the insulin-like growth factor binding system, which plays an essential role in many physiologic processes, such as growth, differentiation, and proliferation of human cells [7]. It can interact with insulin-like growth factor 1 (IGF-1) as well as insulin. The affinity of IGFBP7 for insulin is 500 times higher than other IGF binding proteins. This protein blocks insulin binding to its receptors, reducing the physiological response to insulin, and may indicate the involvement of IGFBP7 in developing insulin resistance, diabetes, and, consequently, cardiovascular disease [7,8]. Elevated concentrations of IGFBP7 were found in patients with HF, both HFpEF and HFrEF. According to the research, IGFBP7 correlates with diastolic dysfunction of the heart in patients with HF. Patients with HFpEF develop diastolic abnormalities due to hypertrophy and interstitial fibrosis, whereas in patients with HFrEF, fibrosis is a replacement process to substitute dead cardiomyocytes. The mechanism behind these findings remains unclear and requires further studies [7]. In the multicenter International Collaborative of NTproBNP-Re-evaluation of Acute Diagnostic Cut-Offs in the Emergency Department (ICON-RELOADED) study, concentrations of N-terminal pro–B-type natriuretic peptide (NTproBNP), high-sensitivity cardiac troponin T (hsTnT), and IGFBP7 were analyzed in 1448 patients presenting with acute dyspnea [9]. In addition to the already established biomarkers, NTproBNP and hsTnT, IGFBP7 allowed for better clinical characterization of patients, had an additive effect in the diagnosis of acute HF decompensation, and allowed for more efficient risk stratification of short-term mortality and HF rehospitalization [9]. What is more, IGFBP7 combined with echocardiography may enhance the diagnosis of acute HF. According to research, IGFBP7 concentrations are associated with a range of cardiac structure and function abnormalities, particularly with left atrium volume index (LAVI)—an indicator of left ventricular filling pressure and myocardial stiffness [10].

Lung ultrasonography (LUS) is a simple and non-invasive diagnostic method increasingly used for rapid diagnosis and monitoring of seriously ill patients with acute heart failure [11]. Assessing the number of B-lines in LUS is simple and repeatable. It is especially useful in diagnosing and monitoring the progress of treatment of acute or exacerbated heart failure [12], even in patients with subclinical symptoms of circulatory decompensation, challenging to diagnose due to obesity [13], or the coexistence of other diseases that may manifest clinical symptoms similar to those in heart failure. LUS is often superior to laboratory and radiological assessment [14]. It does not expose patients to the harmful effects of X-ray radiation. It may support the decision to modify therapy, consequently reducing the risk of rehospitalization and heart failure complications [15,16,17]. However, most studies were conducted in patients with HFrEF, and only a few in HFpEF and HFmrEF; these populations of patients require further investigation.

The aims of our study were as follows:

- To assess the usefulness of LUS as a quick diagnostic method to confirm the cardiac cause of dyspnea in patients with HFpEF and HFmrEF;
- To determine the correlation between the ultrasound image of the lungs and classic (NTproBNP) and new (IGFBP7) biomarkers and echocardiographic markers of HF;
- To assess the importance of B-lines in lung ultrasound and IGFBP7 concentration as a prognostic factor in patients with HFpEF and HFmrEF hospitalized due to exacerbations of HF symptoms.

## 2. Materials and Methods

### 2.1. Study Population

The research was conducted on a group of 143 patients hospitalized between 2018 and 2020, admitted to the Department of Cardiology due to shortness of breath and diagnosed with HFpEF and HFmrEF. Exclusion criteria from the study were pulmonary edema and cardiogenic shock, EF ≤ 40%, and/or severe valvular defect confirmed by echocardiography, non-cardiac causes of dyspnea, such as pneumonia, asthma, chronic obstructive pulmonary disease (COPD), pulmonary emphysema, oncological diseases, or uremia.

In all study patients, baseline characteristics regarding demographics, medical history, concomitant diseases, other causes of dyspnea, diagnostic test results, and currently used pharmacotherapy were collected.

### 2.2. Ethical Issues

The study was approved by the Local Bioethics Committee nr. R-I-002/618/201. Informed consent was obtained from all subjects involved in the study.

### 2.3. Biochemical Evaluation

In all participants, venous blood was collected to obtain basic biochemical parameters, such as blood count, total cholesterol, low-density lipoprotein (LDL) cholesterol, high-density lipoprotein (HDL) cholesterol, triglycerides, glucose, creatinine, coagulation parameters, thyroid stimulating hormone (TSH), C-reactive protein (CRP), and additionally, NTproBNP and IGFBP7. The blood was drawn to clot in a closed system of the Monovette type (SARSTEDT, Nümbrecht, Germany). Biochemical parameters were determined within 2 h after the material’s collection. The blood samples (5 mL) drawn for IGFBP7 determination were left at room temperature for two hours to allow clot formation and then centrifuged at 1000× *g* for 20 min at room temperature. Freshly prepared supernatant serum was frozen and stored at −80 °C until use. The concentration of IGFBP7 was established with commercially available ELISA kit 7 (insulin growth factor binding protein; USCN Life Science Inc., Wuhan, Hubei, China), according to the manufacturer’s instructions. The obtained results are the mean of two almost identical measurements. The glomerular filtration rate was calculated by the Modification of Diet in Renal Disease (MDRD) formula. Blood pressure and anthropometric measurements (height and weight) were performed in all participants. Body mass index (BMI) was calculated as standard. Blood pressure (BP) was measured using the oscillometric method after the participants were seated for at least 10 min. The patients were fasting during the blood collection.

### 2.4. Lung Ultrasonography

All participants, upon admission to the hospital on the first day of hospitalization and again upon discharge, underwent transthoracic lung ultrasound to assess the presence of ultrasound signs of heart failure—the number of B-lines and the presence of fluid in the pleural cavities. The examination was performed using the PHILIPS CX50 (PHIPLIS, Bothell, DC, USA) and AFFINITY 70 (PHIPLIS, Bothell, DC, USA) devices with S5-1 cardiac heads. The number of B-lines and the presence of fluid in the pleural cavities was assessed by placing the transducer in the intercostal areas of the chest on the right and left side, in the subclavicular area in the mid-clavicular line, in the fourth intercostal area in the anterior axillary line, in the sixth intercostal area in the mid-axillary line, in the supra-scapular, interscapular, and subscapular area.

### 2.5. Echocardiography

Echocardiographic examinations were performed on the first day of hospitalization in all subjects following the European Association of Cardiovascular Imaging (EACVI) recommendations, using the Philips devices: iE33 (PHIPLIS, Bothell, DC, USA) and Affinity70 (PHIPLIS, Bothell, DC, USA). Dimensions of the heart chambers, left ventricular wall thickness, left ventricular mass index, heart valve function, wall contractility, and left ventricular ejection fraction (LVEF) were assessed. LVEF was assessed using the Simpson method. The diastolic function of the left ventricle (LV) was assessed based on parameters of inflow through the mitral valve using the pulsed wave Doppler method and the velocity of the basal segment of the intraventricular septum and the lateral wall using the tissue Doppler method, following the 2016 guidelines of the European Society of Cardiology for the diagnosis and treatment of acute and chronic heart failure. Using the M-mode method, right ventricular systolic function was assessed based on the amplitude of tricuspid valve lateral annular motion (TAPSE). The severity of valvular heart disease was assessed according to the currently applicable ESC criteria.

### 2.6. Follow-Up

Two years (±4 months) after hospitalization, a follow-up telephone visit was performed.

### 2.7. Statistical Analysis

The mean values and standard deviations for quantitative variables and the quantitative and percentage distribution for qualitative variables were calculated. Data were presented as means (%) and standard deviation (SD) distributed continuous variables, medians (Me) and interquartile range (IQR) for not normally distributed continuous variables, and as the number (N) of cases and percentages (%) for categorical variables. The statistical significance of differences between the two groups was determined using the t-test (for comparing normal continuous variables) and the U Mann–Whitney test (for comparing non-normal continuous variables). Pearson’s correlation coefficient for categorical variables of normal distribution and Spearman’s correlation coefficient for variables not satisfying the normal distribution criteria were calculated. The comparison of qualitative variables between the groups was performed using the Chi^2^ test. An odds ratio calculator was used for univariate analysis. Multivariate analysis was performed using backward stepwise regression. The statistical analysis was carried out using the Statistica 12.0 PL software (StatSoft Polska Sp. z.o.o., Kraków, Poland). The value of *p* < 0.05 was considered statistically significant.

## 3. Results

### 3.1. Characteristics of the Study Group

The study group comprised 143 patients, with 89 women (63.24%) and 54 men (37.76%). A total of 86 subjects (60.14%) were diagnosed with heart failure with preserved ejection fraction (HFpEF), and 57 subjects (39.86%) had heart failure with mildly reduced ejection fraction (HFmrEF). The general characteristics of the study group are presented in Table 1.

Patients with diagnosed HFpEF had significantly higher systolic blood pressure on admission (150.3 ± 28.3 vs. 138.9 ± 26, *p* = 0.02), were more frequently diagnosed with hypertension (81 (94.19%) vs. 47 (82.46%), *p* = 0.05), compared to patients with HFmrEF. Patients with HFmrEF were significantly more likely to have a history of myocardial infarction (15 (26.32%) vs. 2 (2.33%), *p* < 0.001) and were more likely to smoke (13 (22.81%) vs. 7 (8.24%), *p* = 0.03).

### 3.2. Echocardiography

Patients with EF 41–49% compared to those with EF ≥ 50% had a significantly higher left ventricular end-diastolic dimension (5.2 ± 0.7 cm vs. 4.82 ± 0.51 cm, *p* < 0.001), higher left ventricular mass index (124.88 ± 29.13 vs. 108.81 ± 29.66, *p* = 0.003), larger left atrial dimension (4.6 ± 0.68 vs. 4.36 ± 0.56, *p* = 0.03), higher pulmonary artery systolic pressure (38.8 ± 16.39 vs. 31.1 ± 11.32, *p* = 0.009), and mean pulmonary artery pressure (29.97 ± 9.45 vs. 23.41 ± 8.37, *p* = 0.02), as well as higher right ventricular dimension (4.48 ± 0.84 vs. 3.63 ± 0.86, *p* < 0.001). Right ventricular free wall hypokinesis was also significantly more frequently observed in this group of patients (9 (16.98%) vs. 4 (4.88%), *p* = 0.03).

On the other hand, patients with EF ≥ 50% had a more frequent finding of limited inferior vena cava respiratory motion (75 (93.75%) vs. 40 (81.63%), *p* = 0.04).

No differences in other echocardiographic parameters were found between the patient groups studied; the details are shown in Table 2.

### 3.3. Pharmacological Treatment

Patients with left ventricular ejection fraction (EF) ≥ 50% were more likely to be treated with calcium receptor antagonists (41 (47.67%) vs. 12 (21.05%), *p* = 0.001). In contrast, patients with EF 41–49% were more likely to be treated with an aldosterone antagonist (21 (36.84%) vs. 18 (20.93%), *p* = 0.05), but this difference remained at the limit of statistical significance.

There were no significant differences between the groups regarding the other pharmacotherapy used. Detailed data are shown in Table 3.

### 3.4. Biochemical Tests

Patients with HFpEF compared to those with HFmrEF were more likely to have higher HDL (high-density lipoprotein) cholesterol (46.07 ± 12.7 vs. 40.65 ± 11.85, *p* = 0.01), CRP (C-reactive protein) (4.05 ± 6.44 vs. 3.4 ± 11.2, *p* = 0.04), and higher platelet count (PLT) (226.71 ± 72.03 vs. 198.41 ± 61.41, *p* = 0.02). Patients with HFmrEF had significantly higher NTproBNP levels (649 (256–1421) vs. 845 (431–2229), *p* = 0.04).

NTproBNP levels in the HFmrEF group correlated significantly with NYHA class (r = 0.37, *p* = 0.005), inferior vena cava diameter (r = 0.35, *p* = 0.02), pulmonary artery systolic pressure (r = 0.43, *p* = 0.009), and number of days of hospitalization (r = 0.37, *p* = 0.004).

These two groups had no significant differences in other laboratory parameters, including IGFBP7 levels (2.59 ± 1.85 vs. 2.75 ± 2.05, *p* = 0.66). Detailed data are shown in Table 4.

NTproBNP levels ≥ 1000 pg/mL helped differentiate patients with a severe exacerbation of heart failure symptoms—NYHA class III and IV—with a test sensitivity of 69% and specificity of 58% (AUC = 0.66; 95% CI = 0.55–0.78; *p* = 0.01).

In contrast, IGFBP7 levels determined on admission had no additional diagnostic value for confirming exacerbation of heart failure symptoms (AUC = 0.50; 95% CI = 0.38–0.62; *p* = 0.93).

### 3.5. Lung Ultrasonography

There were no statistically significant differences in the number of B-lines on admission in patients with left ventricular ejection fraction ≥ 50% and EF 41–49% (19.55 ± 17.11 vs. 19.72 ± 17.67, *p* = 0.95).

In contrast, the difference between the number of B-lines on admission and at hospital discharge with a marked reduction during hospitalization reached statistical significance in the group with preserved LV ejection fraction (19.55 ± 11.1 on admission vs. 15.54 ± 13.95 at discharge, *p* < 0.001) and mildly impaired LV ejection fraction (19.72 ± 17.67 on admission vs. 15.91 ± 15.1 at discharge, *p* < 0.001).

In the group of patients with preserved LV ejection fraction, the number of B-lines on admission correlated positively with age (r = 0.22, *p* = 0.03), NYHA class (r = 0.45, *p* < 0.001), pulmonary artery systolic pressure (r = 0.29, *p* = 0.02), and number of days of hospitalization (r = 0.33, *p* = 0.001), while a negative correlation was found with BMI (r = −0.26, *p* = 0.03)—Table 5.

In the group of patients with mildly impaired LV ejection fraction, the number of B-lines on admission correlated positively with NYHA class (r = 0.41, *p* = 0.001), pulmonary artery systolic pressure (r = 0.49, *p* = 0.002), creatinine concentration (r = 0.3, *p* = 0.02), NTproBNP concentration (r = 0.41, *p* = 0.001), and number of days of hospitalization (r = 0.47, *p* = 0.0002)—Table 5.

**Figure 1:**
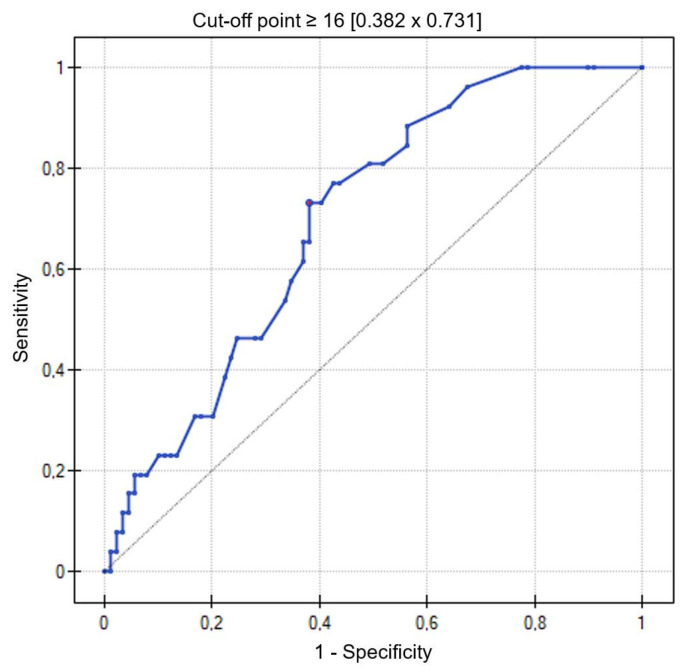
ROC curve for the number of B-lines on lung ultrasonography relative to HF symptom severity—NYHA class on hospital admission.

Based on the ROC curve, the number of B-lines in the LUS ≥ 16 was determined with a sensitivity of—73% and specificity of—62%, indicating exacerbation of heart failure symptoms on admission (AUC = 0.70; 95% CI = 0.59–0.79; *p* = 0.0023) (Figure 1).

### 3.6. Duration of Hospitalization

#### 3.6.1. Patients with Preserved LV Ejection Fraction—EF ≥ 50%

Patients hospitalized for more than three days had a statistically significant higher NYHA class compared to patients hospitalized for a shorter time (2.19 ± 0.6 vs. 1.77 ± 0.57, *p* = 0.003), higher creatinine levels (1.06 ± 0.35 vs. 0.92 ± 0.19, *p* = 0.05), and more B-lines on lung ultrasound on admission (22.75 ± 16.51 vs. 14.89 ± 17.12, *p* = 0.04).

#### 3.6.2. Patients with Mildly Impaired LV Ejection Fraction—EF 41–49%

Patients hospitalized ≥ 3 days had a statistically significant higher NYHA class compared to patients with a shorter hospital stay (2.46 ± 0.54 vs. 2.12 ± 0.45, *p* = 0.02), a wider inferior vena cava (2.06 ± 0.58 vs. 1.62 ± 0.32, *p* = 0.006), higher creatinine levels (1.17 ± 0.19 vs. 0.92 ± 0.51, *p* = 0.04), lower hemoglobin levels (12.64 ± 2.14 vs. 13.88 ± 0.99, *p* = 0.02), and more B-lines on ultrasound on hospital admission (24.54 ± 19.94 vs. 12.05 ± 9.43, *p* = 0.008).

### 3.7. Follow-Up

A telephone visit was performed two years (±4 months) after hospitalization in the Department of Cardiology.

The follow-up period was a mean of 24 months. A total of 55 patients (41.04%) were rehospitalized for heart failure, with 34 (41.46%) in the group with EF ≥ 50% and 21 (40.38%) in the group with EF 41–49%, *p* = NS. A total of 26 patients (19.40%) died, with 20 (24.39%) patients in the group with EF ≥ 50% and 6 (11.54%) in the group with EF 41–49%, *p* = 0.08.

#### 3.7.1. Rehospitalizations

Patients with preserved LV ejection fraction with more advanced age (75.62 ± 6.5 vs. 70.48 ± 12.86, *p* = 0.04) and with diabetes were more frequently rehospitalized (*p* = 0.33).

In the group of patients with mildly impaired LV ejection fraction, those with higher NTproBNP levels were more frequently rehospitalized (median 1726 (IQR 690.6–3368.0) vs. 616 (IQR 335–1229), *p* = 0.003).

The number of B-lines on lung ultrasound did not affect the rehospitalization rate.

#### 3.7.2. Mortality

The analysis of deaths was performed on the whole study group due to the size of the group and the number of these events. Patients who died at 24 months follow-up were older than the surviving ones (82.1 ± 6.8 vs. 70.02 ± 10.14, *p* < 0.001), had higher creatinine levels (1.24 ± 0.48 vs. 0.98 ± 0.32, *p* < 0.001), more advanced heart failure symptoms according to the NYHA classification (2.64 ± 0.55 vs. 2.02 ± 0.56, *p* < 0.001), a higher number of B-lines on ultrasound, both on hospital admission (28.08 ± 16.57 vs. 16 ± 15.5, *p* < 0.001) and at discharge (21.52 ± 14.42 vs. 14.18 ± 12.81 *p* = 0.01), lower LDL cholesterol (90.5 ± 39.1 vs. 113.3 ± 46.4, *p* = 0.02) and triglycerides (97.89 ± 32.25 vs. 122.56 ± 55.8, *p* = 0.03), lower hemoglobin levels (12.34 ± 1.37 vs. 13.54 ± 1.65, *p* < 0.001), and longer hospitalization time (mean 6.15 ± 3.52 vs. 4.38 ± 2.24, *p* = 0.002).

**Figure 2:**
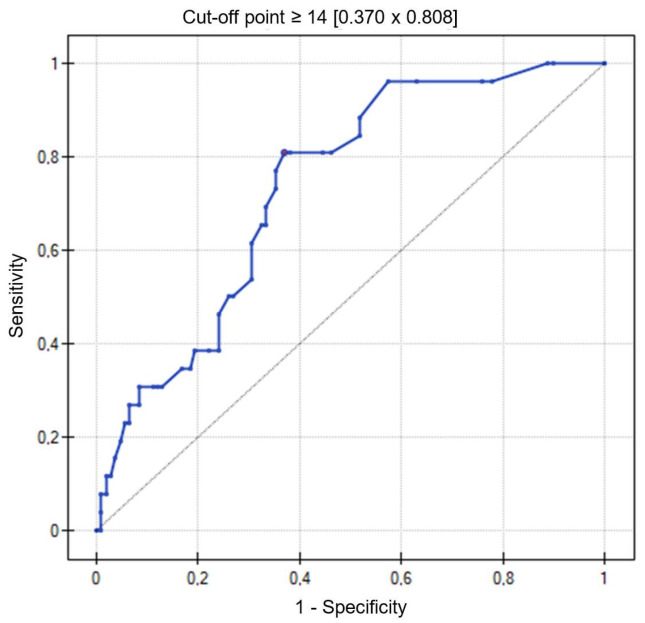
ROC curve for the number of B-lines on lung ultrasound relative to the risk of death during the 24-month follow-up period.

In the study group of patients, the number of B-lines on lung ultrasound ≥14 was determined as a cut-off point (AUC = 0.73, 95% CI = 0.64–0.83, *p* = 0.0002; sensitivity 81%, specificity 63%) indicating an increased risk of death during the follow-up period (Figure 2).

### 3.8. Multivariate Analysis

**Figure 3:**
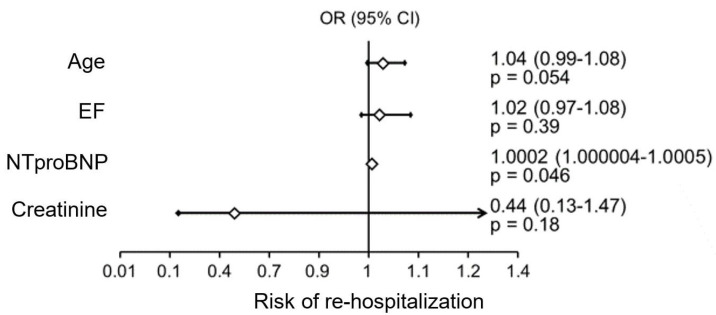
Factors influencing the risk of rehospitalization.

**Figure 4:**
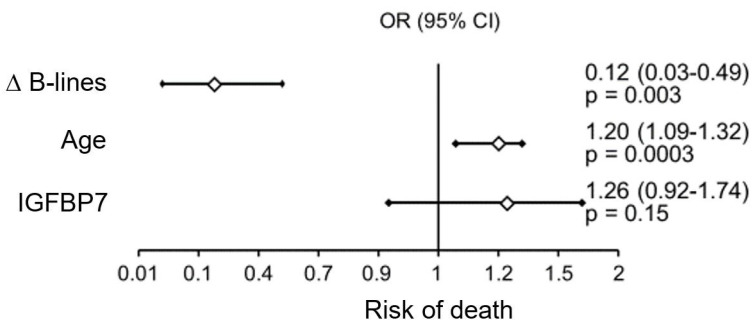
Factors influencing the risk of death during follow-up.

Multivariate analysis was performed by stepwise regression in the entire study population, including the following parameters: number of B-lines and ∆ B-lines, age, LV ejection fraction, creatinine concentration, GFR, NTproBNP, IGFBP7, hemoglobin concentration, and total cholesterol and LDL-cholesterol fraction, which showed the following:

- A significant independent factor influencing the risk of rehospitalization was NTproBNP levels (Figure 3);
- Factors significantly impacting mortality in the study patient population were patient age and the difference between the number of B-lines on ultrasound on admission and at hospital discharge (Figure 4).

IGFBP7 levels had no significant effect on the duration of hospitalization, risk of rehospitalization, or mortality in the patient population studied.

## 4. Discussion

As mentioned in the introduction section, populations of patients with HFpEF and HFmrEF, compared to those with HFrEF, were significantly less studied. There are problems with their proper diagnosis, particularly HFpEF, which encouraged us to conduct our study on this group of patients. The effective management of patients with heart failure exacerbation requires early diagnosis, identification of underlying and reversible causes, and implementation of effective therapy as soon as possible. All of these factors are associated with a better in-hospital and short-term prognosis [18].

The advantages of ultrasound in acute cardiac decompensation are well-recognized and documented in many studies. According to the research, LUS is more sensitive than chest radiography in detecting cardiogenic pulmonary edema. Therefore, this modality should be considered an additional imaging modality in assessing patients with dyspnea [19]. In patients with moderate to high clinical probability of acute cardiogenic pulmonary edema, ultrasound demonstrating the presence of B-lines can be used to enhance the emergency physician’s initial clinical diagnosis of pulmonary edema. A negative ultrasound examination can virtually exclude features of stasis in the pulmonary circulation in patients with a low clinical probability of cardiogenic pulmonary edema. The sensitivity of B-lines on ultrasound for diagnosing cardiogenic pulmonary edema is 94.1%, and the specificity is 92.4% [20]. Visualization of three or more B-lines in two or more intercostal spaces bilaterally should be considered diagnostic [20,21]. In contrast, physical examination and chest radiography have sensitivities of only 62% and 57%, respectively, and specificities of 68% and 89% for diagnosing pulmonary edema [22,23]. Most of the data in the literature concerns the use of pulmonary ultrasonography in heart failure patients with HFrEF. Single studies have been conducted among heart failure patients with HFpEF [24,25]. According to our knowledge, there are no published studies on the use of pulmonary ultrasonography in the diagnosis of HFmrEF. In our study, the assessment of the number of B-lines in lung ultrasonography on admission was characterized by a good diagnostic value indicating exacerbation of heart failure symptoms, both in patients with HFpEF and HFmrEF. There were no statistically significant differences in the number of B-lines on LUS on admission in patients with EF ≥ 50% and with EF 41–49%. In contrast, the difference between the number of B-lines on admission and at hospital discharge with a marked reduction during hospitalization reached statistical significance in both groups with HFpEF and HFmrEF. Similar observations have also been made by other authors [26,27]. The number of B-lines is thought to decrease during acute heart failure (AHF) treatment; therefore, LUS is potentially valuable for monitoring the efficacy of HF exacerbation treatment.

During the 24-month follow-up period, a high rehospitalization rate of 41.04% was recorded, which is consistent with the European data, in which approximately 50% of patients hospitalized due to AHF will require rehospitalization within 12 months [28]. In our study, the number of B-lines on lung ultrasound did not affect the rehospitalization rate. Similar results were obtained by Mhanna M. et al.; however, they also confirmed the usefulness of providing outpatient diuretic therapy under the guidance of LUS. Such management reduced the number of urgent visits due to the severity of HF symptoms [29]. In other studies, a high number of B-lines before hospital discharge was an independent predictor of hospital readmission for HF, both within 30 days of discharge [30] and within 6 months [27,31]. The absence or low number of B-lines identified a subgroup with an extremely low risk of rehospitalization for HF decompensation [27]. Other researchers showed that the number of B-lines was only associated with the incidence of a composite endpoint at short-term follow-up, while it did not affect the outcomes at 60-day and 180-day follow-up [32]. In contrast, Palazzuoli A. et al. obtained different results; they observed that the ∆ (difference between the number of B-lines at admission and hospital discharge) of B-lines was associated with the incidence of the composite endpoint of death and rehospitalization at the 6-month follow-up [25]. In our study, ∆ B-line did not affect the risk of rehospitalization, but it was a significant independent factor affecting the risk of death at the 24-month follow-up.

In our study population, patients who died during the 24-month follow-up had significantly more B-lines in the LUS, both on hospital admission and at discharge. Similar results were obtained by Palazzuoli A. et al. in a 6-month follow-up [24,25], both in patients with HFpEF and HFrEF, as well as by Platz E. et al. [33]. Other investigators confirmed the importance of B-lines as an independent prognostic factor of worsening heart failure in acute myocardial infarction [34]. In contrast, Marini C et al. did not show that pharmacological treatment of AHF under pulmonary ultrasound guidance had an impact on patient mortality at 90-day follow-up [35]. Also, other investigators did not confirm that the severity of lung ultrasound lesions influenced the prognosis of patients hospitalized for cardiovascular decompensation, while NTproBNP levels proved to be an independent prognostic factor [36]. According to other authors, both elevated NTproBNP levels and B-line counts ≥15 were associated with the risk of death and hospitalization for heart failure [37]. In our study, the number of B-lines ≥14 with good test sensitivity indicated an increased risk of death during the follow-up period. In contrast, NTproBNP levels with a cut-off point ≥ 982.1 pg/mL and IGFBP7 levels ≥2.52 ng/mL had a lower sensitivity in estimating the risk of death in the patient population studied. Due to the discrepancy in the results obtained, this issue requires further study.

In our study, the number of B-lines also influenced the duration of hospitalization. Both HFpEF and HFmrEF patients hospitalized for more than three days had statistically significantly more B-lines on LUS on hospital admission and, therefore, required prolonged diuretic therapy. According to the joint statement of the European Society of Cardiology and the Heart Failure Association, 15–30 lines represent moderate stasis, and more than 30 B-lines at 28 points indicate severe congestive changes [38]. In our study, patients who required prolonged hospitalization had >20 B-lines on admission. In previous studies, it has been observed that patients who were dehydrated under LUS guidance had a faster clinical improvement [39], although according to other research, quite a long period (median 13.5 days) is needed to reduce B-lines on lung ultrasound [32]. In addition, it should be noted that our patients did not receive either flosine or sacubitril/valsartan for heart failure therapy (the inclusion period of the study was 2018–2019). A study is currently underway to assess the efficacy of intensifying HF treatment under pulmonary ultrasound guidance in the era of pharmacotherapy with SGLT-2 inhibitors (sodium–glucose cotransporter-2 inhibitors) and ARNI (angiotensin receptor blocker–neprilysin inhibitors) [40].

Studies assessing the diagnostic value of lung ultrasonography versus natriuretic peptides for identifying AHF in patients with dyspnea have reported variable results in different cohorts [21,41,42]. In our study, we found that significantly higher NTproBNP levels in patients with EF 41–49% (HFmrEF) correlated with NYHA class, inferior vena cava diameter, pulmonary artery systolic pressure, and number of days of hospitalization. In contrast, no association was found between NTproBNP levels and the number of B-lines in lung ultrasound on admission, nor with IGFBP7 levels. In another study, multiple bilateral B-lines in AHF correlated well with natriuretic peptide levels [42]. Moreover, other investigators have confirmed a significant correlation between NTproBNP levels and the severity of lung ultrasound lesions on hospital admission and discharge, which had prognostic significance [24]. In our study group, NTproBNP concentration was a factor influencing the risk of rehospitalization but not the risk of death or prolonged hospitalization.

Previous studies have suggested that the IGFBP7 protein may be a good biomarker in heart failure. According to the research, baseline higher IGFBP7 levels in patients with HFpEF were associated with an increased risk of a primary composite endpoint, including all-cause mortality and HF events. The mechanism behind these results remains to be determined. It may be due to myocardial fibrosis, which is thought to be an important factor in the development of HFpEF. Previous studies have observed a significant correlation between IGFBP7 levels and interstitial collagen deposition [43,44]. Our study found no significant differences in IGFBP7 levels between patients with EF ≥ 50% and EF 41–49%. What is more, IGFBP7 concentration determined on hospital admission was shown to have no additional diagnostic value for confirming the exacerbation of heart failure symptoms. IGFBP7 levels were also without significant effect on the duration of hospitalization, risk of rehospitalization, and mortality in the study patient population. Data on the role of IGFBP7 as a diagnostic and prognostic marker in heart failure are divergent. An earlier study by Lisowska et al. found no statistically significant differences between patients with EF > 50% and EF < 50% [45]. Other investigators have also failed to demonstrate an association between IGFBP7 levels and left ventricular mass, volume, and systolic function in heart failure with reduced LV ejection fraction [43]. In contrast, Barosso et al. observed that IGFBP7 may be a better early predictor of heart failure than NTproBNP [46].

Mortality in the study group was 19.4 percent at 24-month follow-up. Of note, there were significantly more deaths in the group of patients with HFpEF compared to those with HFmrEF. Our results differ from the literature data. Data from the European registry show that annual mortality differed significantly between patients with HFrEF and HFpEF (8.8% vs. 6.3%), and it was 7.6% in the HFmrEF group [5]. In the Chinese population, the difference in 5-year mortality between HFmrEF and HFpEF was 18.1 vs. 13.4%, respectively [47]. In contrast, other researchers have shown that among patients hospitalized for heart failure, 5-year survival was similar in HFpEF and HFmrEF [48,49]. Quiroz et al. reported similar 30-day and 1-year mortality in these patient subpopulations [50]. In general, survival is thought to be higher in HFpEF than in HFrEF, but most observational studies indicate that this difference is slight [1].

In conclusion, lung ultrasound should be used as an adjunct to the classic physical examination in order to make a correct and rapid diagnosis of heart failure exacerbation. Using LUS in daily clinical practice may also improve the monitoring and treatment of patients with HF. Notably, a higher number of B-lines on LUS at hospital discharge may help identify patients with heart failure with a worse prognosis. In contrast, IGFBP7 levels have not been shown to have diagnostic or prognostic significance in the patient population studied. However, this issue requires further study due to the discrepancy between the results obtained and the literature data.

The limitation of our research is the small size of the study group, which was caused by patient recruitment problems during the COVID-19 pandemic. Further research on this topic should be conducted. Moreover, one of the inclusion criteria—left ventricular diastolic dysfunction—was assessed according to guidelines from 2016 [20], as the 2021 European guidelines were not available at the beginning of the study. Moreover, the IGFBP7 cut points have not been prospectively validated yet and probably vary from population to population.

Finally, the results regarding higher follow-up mortality in the HFpEF group should be interpreted with caution. The lack of data on the cause of death does not allow an assessment of the impact of comorbidity on increased mortality in this subpopulation of patients.

## 5. Conclusions

Lung ultrasonography provides additional diagnostic value in patients with HFpEF or HFmrEF and an exacerbation of heart failure symptoms. The number of B-lines ≥14 may indicate an increased risk of death. IGFBP7 levels had no significant effect on hospitalization duration, rehospitalization risk, and mortality in the study patient population; its role in HFpEF and HFmrEF requires further studies.

## Figures and Tables

**Table 1:** Characteristics and comparison of the study groups with HFpEF and HFmrEF.

	Study Group (n = 143)	EF ≥ 50% (n = 86)	EF 41–49% (n = 57)	*p*
Age, years	72.28 ± 10.54	72.28 ± 10.79	72.28 ± 10.26	0.99
Sex (female, %)	89 (62.24%)	59 (68.60%)	30 (52.63%)	0.08
Left ventricle ejection fraction (%)	51.04 ± 6.5	55.23 ± 4.71	44.70 ± 2.38	
NYHA Class II	114 (79.7%)	74 (86.05%)	41 (71.93%)	0.08
NYHA Class III, IV	29 (20.3%)	12 (13.95%)	16 (28.07%)	0.08
Systolic BP, mmHg	145.7 ± 27.9	150.3 ± 28.3	138.9 ± 26	0.02
Diastolic BP, mmHg	83.4 ± 15.4	84.7 ± 14.9	81.4 ± 16.1	0.21
Heart rate on admission (u/min)	80.2 ± 20.6	79.2 ± 20	81.6 ± 21.6	0.49
BMI, kg/m^2^	30.02 ± 5.89	29.38 ± 5.43	31.01 ± 6.5	0.15
BSA (m^2^)	1.92 ± 0.24	1.91 ± 0.23	1.93 ± 0.26	0.66
Length of hospitalization, days	4.78 ± 2.73	4.62 ± 2.49	5.04 ± 3.07	0.37
Atrial fibrillation, n (%)	53 (37.06%)	28 (32.56%)	25 (43.86%)	0.22
Hypertension, n (%)	128 (89.51%)	81 (94.19%)	47 (82.46%)	0.05
Diabetes t.2, n (%)	56 (39.16%)	29 (33.72%)	27 (47.37%)	0.12
Hyperlipidemia, n (%)	103 (72.03%)	60 (69.77%)	43 (75.44%)	0.57
History of myocardial infarction, n (%)	17 (11.89%)	2 (2.33%)	15 (26.32%)	<0.001
Smoking in the present, n (%)	20 (14.08%)	7 (8.24%)	13 (22.81%)	0.03

Abbreviations: BMI—body mass index; BP—blood pressure; BSA—body surface area, EF—ejection fraction.

**Table 2:** Characterization of echocardiographic parameters.

	EF ≥ 50% (n = 86)	EF 41–49% (n = 57)	*p*
LVDd (cm)	4.82 ± 0.51	5.2 ± 0.7	<0.001
IVSd (cm)	1.2 ± 0.52	1.18 ± 0.19	0.72
PWd (cm)	1.05 ± 0.13	1.08 ± 0.13	0.16
RVID (cm)	2.98 ± 0.47	3.11 ± 0.5	0.13
LVMI (g/m^2^)	108.81 ± 29.66	124.88 ± 29.13	0.003
Ao (cm)	3.43 ± 0.41	3.46 ± 0.39	0.67
LA (cm)	4.36 ± 0.56	4.6 ± 0.68	0.03
Pulmonary artery systolic pressure (mmHg)	31.1 ± 11.32	38.8 ± 16.39	0.009
Mean pulmonary artery pressure (mmHg)	23.41 ± 8.37	29.97 ± 9.45	0.02
Right ventricular dimension (cm)	3.63 ± 0.86	4.48 ± 0.84	<0.001
Inferior vena cava diameter (cm)	1.82 ± 1.85	1.89 ± 0.54	0.79
Stiff inferior vena cava (yes, %)	75 (93.75%)	40 (81.63%)	0.04
Hypokinetic right ventricle (yes, %)	4 (4.88%)	9 (16.98%)	0.03

Abbreviations: Ao—ascending aorta dimension; IVSd—interventricular septal thickness at end-diastole; LA—left atrial dimension; LVDd—left ventricular end-diastolic dimension; LVMI—left ventricular mass index to body surface area; PWd—posterior wall thickness at end-diastole; RVID—diastolic right ventricular internal diameter; stiff inferior vena cava—no respiratory variation > 50% of inferior vena cava diameter.

**Table 3:** Pharmacological treatment.

	EF ≥ 50% (n = 86)	EF 41–49% (n = 57)	*p*
Anticoagulant treatment, n (%)	41 (47.67%)	32 (56.14%)	0.40
Acetylsalicylic acid, n (%)	25 (29.07%)	19 (33.33%)	0.71
Angiotensin-converting enzyme inhibitor, n (%)	62 (72.09%)	46 (82.14%)	0.23
Sartan, n (%)	17 (19.77%)	8 (14.04%)	0.50
Beta-blocker, n (%)	73 (84.88%)	51 (89.47%)	0.46
Aldosterone receptor antagonist, n (%)	18 (20.93%)	21 (36.84%)	0.05
Calcium antagonist, n (%)	41 (47.67%)	12 (21.05%)	0.001
Diuretic, n (%)	61 (70.93%)	47 (82.46%)	0.16
Statin, n (%)	71 (82.56%)	41 (71.93%)	0.15
Antiarrhythmic drug, n (%)	12 (13.95%)	9 (15.79%)	0.81
Oral antidiabetic drug, n (%)	21 (24.42%)	21 (37.50%)	0.13
Insulin, n (%)	7 (8.14%)	4 (7.02%)	1.00

Abbreviation: EF—ejection fraction.

**Table 4:** Biochemical results.

	EF ≥ 50% (n = 86)	EF 41–49% (n = 57)	*p*
NTproBNP (Me, IQR) (pg/mL)	649 (256–1421)	845 (431–2229)	0.04
Creatinine (mg/dL)	1.00 ± 0.3	1.075 ± 0.44	0.24
eGFR (mL/min/1.73 m^2^)	71.22 ± 23.84	74.26 ± 31.07	0.51
Total cholesterol (mg/dL)	173.42 ± 50.76	157.84 ± 58.49	0.10
LDL cholesterol (mg/dL)	111.17 ± 43.89	99.93 ± 48.65	0.16
HDL cholesterol (mg/dL)	46.07 ± 12.7	40.65 ± 11.85	0.01
Triglycerides (mg/dL)	119.80 ± 52.5	111.71 ± 51.44	0.37
Glucose (mg/dL)	109.05 ± 37.37	111.75 ± 37.47	0.68
CRP (Me, IQR)	4.05 ± 6.44	3.4 ± 11.2	0.04
PT (s)	21.65 ± 12.13	23.015 ± 15.68	0.67
APTT (s)	31.92 + 12.66	30.98 ± 15.61	0.83
INR	1.67 ± 0.95	1.71 ± 1.07	0.87
Fibrinogen (mg/dL)	475.06 ± 257.71	331.63 ± 86.93	0.16
Hb (g/dL)	13.32 ± 1.57	13.105 ± 1.88	0.46
WBC (×10^9^/L)	7.56 ± 2.00	6.85 ± 2.28	0.05
PLT (×10^9^/L)	226.71 ± 72.03	198.411 ± 61.41	0.02
IGFBP-7 (ng/mL)	2.59 ± 1.85	2.75 ± 2.05	0.66

Abbreviations: APTT—activated partial thromboplastin time; CRP—C-reactive protein; eGFR—estimated glomerular filtration rate; HDL—high-density lipoprotein; Hb—hemoglobin concentration; IGFBP7—insulin-like growth factor binding protein 7; INR—international normalized ratio; LDL—low-density lipoprotein; NTproBNP—N-terminal fragment brain natriuretic peptides; PLT—platelets; PT—prothrombin time; WBC—white blood cells.

**Table 5:** Correlations of B-lines on admission. NS—Not significant.

	Patients with HFpEF	Patients with HFmrEF
Age	r = 0.22, *p* = 0.03	NS
NYHA class	r = 0.45, *p* < 0.001	r = 0.41, *p* = 0.001
Creatinine concentration	NS	r = 0.3, *p* = 0.02
NTproBNP concentration	NS	r = 0.41, *p* = 0.001
Pulmonary artery systolic pressure	r = 0.29, *p* = 0.02	r = 0.49, *p* = 0.002
Number of days of hospitalization	r = 0.33, *p* = 0.001	r = 0.47, *p* = 0.0002
BMI	r = −0.26, *p* = 0.03	NS

## Data Availability

We do not plan to post the raw research data in the field repository, because our study is planned as a multiyear project and the research data obtained will be used in a series of scientific publications developed by several researchers from our university over the course of up to 5 years of the study (long-term follow-up of the study group is planned). The data will be made available at the request of the scientist, during collaborations with other scientists.
